# P-1446. Impact of Social Determinants of Health on Infectious Outcomes in Patients Undergoing Amputation

**DOI:** 10.1093/ofid/ofae631.1618

**Published:** 2025-01-29

**Authors:** Patrick Passarelli, Mike Sportiello, Joshua Geiger, Theresa Tonozzi, Karina Newhall, Alexandra Yamshchikov

**Affiliations:** University of Rochester Medical Center, Rochester, New York; University of Rochester Medical Center, Rochester, New York; University of Rochester Medical Center, Rochester, New York; Lake Erie College of Osteopathic Medicine - Elmira, Elmira, New York; University of Rochester Medical Center, Rochester, New York; University of Rochester School of Medicine and Dentistry, Rochester, New York

## Abstract

**Background:**

Studies have demonstrated the impact of SDOH on outcomes following vascular surgery, but few have focused on risk factors for infectious complications following major amputation.

Odds Ratios (Confidence Intervals) for Infectious Outcomes Based on Social Determinants of Health

**Figure:**
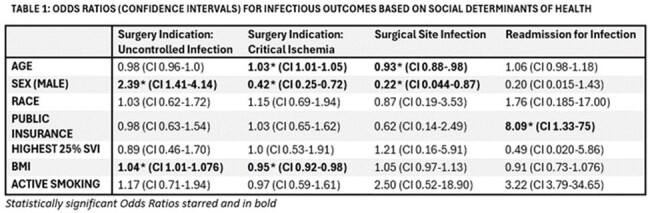

**Methods:**

Local data for the Society Vascular Surgery Vascular Quality Initiative, a multicenter registry of quality improvement, were queried for demographics, clinical, procedure, and outcomes of 371 patients with amputation as index procedure 6/1/2015 to 7/31/2023. Zip codes were used to derive CDC/ATSDR Social Vulnerability Index (SVI). The impact of SDOH and SVI on surgical outcomes with a focus on infection was evaluated by logistical regression with R v4.3.2. Primary outcomes for univariate analysis included surgical site infection (SSI), readmission for infection, composite infection-related outcome, organ system complications during index admission, mortality at 30 days, 1 year, 2 years, and 5 years, and composite surgical adverse outcome inclusive of infectious and non-infectious complications. Significant variables were selected for multivariate regression.

Odds Ratios (Confidence Intervals) Additional Factors and Surgical Outcomes Based on Social Determinants of Health

**Figure:**
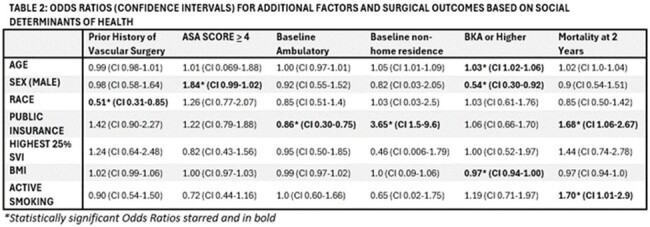

**Results:**

SDOH correlated with multiple parameters in univariate analysis (Fig 1, 2). Male patients had higher ASA score and uncontrolled infection as indication for amputation but were less likely to incur an SSI, or require above trans-metatarsal amputation. Publicly insured patients were more likely to be wheelchair bound or bedridden, or residing in a facility preoperatively, and had the highest risk of readmission for infection (OR=8.09), along with a higher risk of 2 year mortality. Patients with ongoing tobacco use had increased mortality at 1 (OR=1.9) and 2 years (OR=1.7). In multivariate analysis, male gender was associated with SSI (OR 0.25 CI 0.06-0.96). Composite infectious complication was associated with emergent procedure status (OR 2.96 CI 1.22-6.97). There was no significant difference in survival for SDOH derived subgroups (Fig 1) or composite surgical adverse outcome.

Kaplan-Meier Survival Analysis for subgroups based on SDOH parameters

**Figure:**
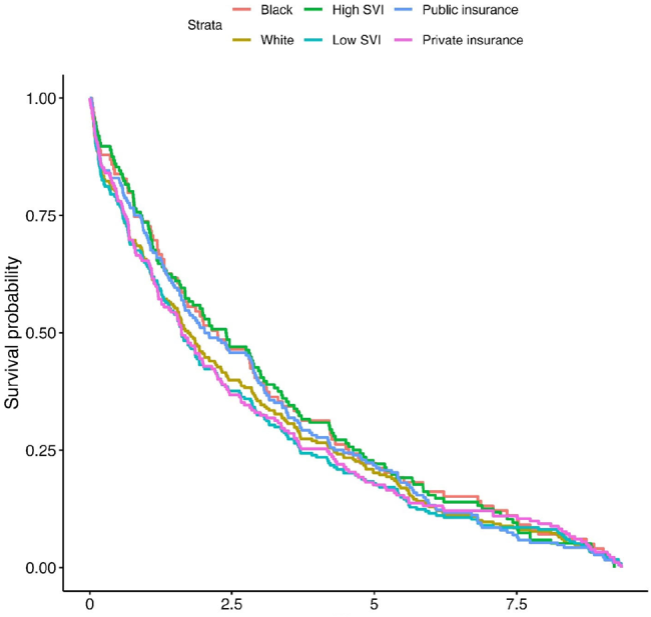

**Conclusion:**

Association between demographic, clinical, and peri-operative complications, with focus on infection, provides opportunity to identify high-risk patients, and serves as a framework for additional interventions, such as close post-operative follow up, and robust involvement of social work and care management teams.

**Disclosures:**

**All Authors**: No reported disclosures

